# Evaluation of venetoclax and hypomethylating agent combination therapy in acute myeloid leukemia: a real-life, single-center experience

**DOI:** 10.1186/s12885-026-16560-8

**Published:** 2026-07-16

**Authors:** Kübra Oral, Ayşe Uysal

**Affiliations:** Department of Hematology, Faculty of Medicine Hospital, Firat University, Elazig, 23119 Turkey

**Keywords:** Acute Myeloid Leukemia, Venetoclax, Survival

## Abstract

**Aim:**

The use of venetoclax in combination with hypomethylating agents (HMAs) has become a standard treatment approach for both newly diagnosed and relapsed/refractory (R/R) AML patients who are ineligible for intensive chemotherapy. We aimed to share the real-life data of this combination therapy.

**Method:**

This retrospective study included 38 patients with newly diagnosed or R/R AML who received at least one cycle of venetoclax-HMA combination therapy between July 2018 and August 2025. Response status, survival rates, and the incidence of hematological adverse events were analyzed.

**Results:**

Twelve patients (31.6%) were treatment-naive, while the remaining 26 (68.4%) were R/R AML. The median age of all patients was 67, and 57.9% were male. 23.7% of the patients were in the poor-risk group. The overall response rate (ORR) was 73.7% in the entire cohort, and 83.3% and 69.2% in the first-line and R/R groups, respectively. No statistically significant difference in ORR was observed between the newly diagnosed and R/R groups (*p* = 0.453); however, given the small sample sizes — particularly in the newly diagnosed arm (*n* = 12) — this study was insufficiently powered to detect or exclude a clinically meaningful difference between groups. The restricted mean progression-free survival (RMST-PFS) was 18.35 months (95% CI 15.16–21.22), and the restricted mean overall survival (RMST-OS) was 21.12 months (95% CI 19.03–22.86). As adverse events, grade 3 or higher neutropenia was 39.5% and thrombocytopenia was 31.6%.

**Conclusion:**

In this single-center real-world experience, venetoclax-HMA combination therapy demonstrated response rates and a toxicity profile consistent with those reported in larger studies, supporting its feasibility in routine clinical practice for AML patients ineligible for intensive chemotherapy.

## Key points

• In this real-world single-center cohort, venetoclax combined with hypomethylating agents yielded an overall response rate of 73.7%, which is consistent with rates reported in larger published studies of this regimen.

• RMST-PFS was 18.35 months and RMST-OS was 21.12 months indicating a meaningful clinical benefit in patients who are not eligible for intensive chemotherapy.

• Although side effects such as low blood counts were common, the treatment was generally safe and manageable in real-life practice.

## Introduction

Acute myeloid leukemia (AML) is a heterogeneous malignancy resulting from the proliferation of clonal immature myeloid cells in the peripheral blood and bone marrow, leading to the failure of normal hematopoiesis [1]. The median age at diagnosis is 68, and its incidence increases with age. Significant differences in survival are observed across various age groups; while the estimated 5-year survival rate is approximately 50% in younger patients, it is less than 10% in those over 60 years of age [2].

The standard treatment for acute myeloid leukemia is intensive chemotherapy containing cytarabine and anthracyclines, followed by allogeneic hematopoietic stem cell transplantation (allo-HSCT). However, intensive therapies are not recommended for elderly patients, those with poor performance status, or those with organ dysfunction due to increased morbidity and early mortality [3]. Therefore, less intensive treatment regimens, including hypomethylating agents (HMAs) and low-dose cytarabine (LDAC), have been utilized for this patient group. With low-intensity therapies, complete response rates, including those with incomplete hematologic recovery, remain approximately 30% with HMAs and 11–19% with LDAC [4, 5].

Proteins belonging to the B-cell lymphoma-2 (Bcl-2) family play a crucial role in the apoptotic response [6]. It has been determined that the expression of Bcl-2 family proteins is increased in acute myeloid leukemia blasts and that these blasts are dependent on the Bcl-2 protein for their survival. Furthermore, it has been demonstrated that AML patients with higher Bcl-2 expression have lower response rates to chemotherapy and shorter survival [7]. Venetoclax (VEN), a potent and selective inhibitor of B-cell lymphoma-2, has been used in hematological malignancies both as monotherapy and in combination with other agents [8, 9]. In the phase 3 VIALE-A study, which evaluated treatment-naive AML patients ineligible for standard induction therapy, the VEN-azacitidine combination achieved a CR + CRi rate of 66% and was shown to significantly increase overall survival (OS) [10].

Despite the growing body of evidence supporting venetoclax-HMA therapy in AML, real-world outcomes may vary across healthcare systems, patient populations, supportive care practices, and transplantation strategies. Therefore, additional real-world data remain valuable for evaluating the generalizability of clinical trial findings and treatment outcomes in routine clinical practice. We aimed to describe the response rates, survival outcomes, and toxicity profile of venetoclax-HMA combination therapy in a real-world cohort of ineligible for intensive chemotherapy AML patients treated at a single center.

## Materials and methods

### Study design and patient selection

In this retrospective study, 38 adult patients who were ineligible for intensive chemotherapy and received at least one cycle of HMA-VEN combination therapy for either newly diagnosed or R/R AML between July 2018 and July 2025 were included. The study cohort represents a selected subgroup of AML patients treated at our institution during the study period. Only patients who were considered unsuitable for intensive chemotherapy and who received venetoclax in combination with a hypomethylating agent were eligible for inclusion. Patients treated with intensive induction chemotherapy, alternative salvage regimens, or supportive care alone were not included in the present analysis. Therefore, the cohort size reflects the eligibility criteria of the study rather than the overall AML population treated at our center. Patient data were obtained by screening medical and electronic records.

### Data collection

The patients’ demographic characteristics at the start of treatment, comorbidity status, cytogenetic risk status, baseline bone marrow blast percentage, line of VEN therapy, number of cycles, the specific HMA used in the combination, status of allo-HSCT after VEN, relapse status after allo-HSCT, VEN-related hematological adverse events, use of antifungal prophylaxis, treatment responses, and survival status/durations were retrospectively screened and recorded.

The hypomethylating agent was administered as either azacitidine 75 mg/m²/day subcutaneously for 7 days or decitabine 20 mg/m²/day intravenously for 5 days, according to the physician’s preference. Both azacitidine and decitabine were administered in 28-day cycles. Venetoclax was administered orally once daily. To prevent tumor lysis syndrome, the venetoclax dose was ramped up as 100 mg on day 1, 200 mg on day 2, and 400 mg on day 3. For tumor lysis prophylaxis, patients were given oral and intravenous hydration along with a uric acid-reducing agent. In the event of hematological toxicity, the venetoclax dose was reduced or discontinued. When antifungal prophylaxis was administered, posaconazole was the agent used in all cases. During posaconazole administration, the venetoclax dose was reduced to 100 mg/day as recommended. Prophylaxis was generally applied according to the clinicians’ discretion until a stable neutrophil count recovery was achieved.

Response assessment was performed based on bone marrow and hemogram parameters. Complete Remission (CR) was defined as a bone marrow blast count of < 5%, the absence of circulating blasts and blasts with Auer rods, no evidence of extramedullary disease, an absolute neutrophil count (ANC) of ≥ 1,000/µL, and a platelet count of ≥ 100,000/µL. CR with incomplete hematologic recovery (CRi) was defined as meeting all CR criteria except for an ANC of < 1,000/µL and/or a platelet count of < 100,000/µL. Partial Remission (PR) was defined as a bone marrow blast percentage between 5% and 25%, or at least a 50% reduction from the initial blast percentage. Refractory disease was defined as the failure to achieve a response after two cycles of treatment. Composite complete remission (cCR) was accepted as CR + CRi, and the overall response rate (ORR) was accepted as cCR + PR [11].

Overall survival (OS) was calculated from the date of initiation of venetoclax treatment until the date of death from any cause or the last follow-up date. Progression-free survival (PFS) was calculated from the date of remission achievement following venetoclax treatment until relapse, death from any cause, or last follow-up. Only patients who achieved remission after venetoclax-based therapy were eligible for PFS analysis; patients with refractory disease or those who did not achieve remission were excluded.

The severity of adverse events was graded according to the National Cancer Institute Common Terminology Criteria for Adverse Events (CTCAE), version 5 [12].

### Statistical analysis

Statistical analyses were performed using “IBM SPSS Statistics for Windows, Version 25.0 (Statistical Package for the Social Sciences, IBM Corp., Armonk, NY, USA)” and the survRM2 package. Descriptive statistics are presented as n (%) for categorical variables and as median (min–max) for continuous variables. The distribution of continuous variables was assessed for normality by visual inspection and the Shapiro–Wilk test. Continuous variables were compared between two independent groups using the Mann–Whitney U test. Categorical variables were compared using the chi-square test or Fisher’s exact test, as appropriate, with Fisher’s exact test applied when expected cell frequencies were low. In survival analyses, survival curves were initially obtained using the Kaplan–Meier (KM) method. However, due to the high censoring rates and the fact that median survival time could not be reached in some subgroups, the Restricted Mean Survival Time (RMST) approach was preferred for intergroup survival comparisons. RMST is defined as the area under the Kaplan–Meier survival curve up to a pre-specified time horizon (τ) k and provides a clinically interpretable measure of mean survival without requiring the proportional hazards assumption. In this study, the truncation time for RMST calculations was set at τ = 24 months. In this study, the truncation time for RMST calculations was set at τ = 24 months. This time horizon was selected because it represents a clinically meaningful follow-up period for patients receiving venetoclax–HMA therapy for AML and because the available follow-up data in our cohort allowed for more reliable estimation and interpretation of survival outcomes up to this time point. For comparisons between two groups, the 24-month RMST mean values and 95% confidence intervals (CI) were reported for each group; additionally, RMST differences (comparison group – reference group) along with their respective 95% CIs and *p*-values were calculated. The statistical significance of RMST differences was evaluated using the survRM2 package. A *p*-value of < 0.05 was considered statistically significant.

No formal sample size or power calculation was performed because of the retrospective nature of the study. Accordingly, subgroup analyses were considered exploratory only, and the available sample sizes were insufficient to support meaningful comparisons between newly diagnosed and relapsed/refractory AML patients.

## Results

A total of 38 patients were included in the study. With respect to disease subtype, 17 patients (44.7%) had de novo AML, 17 (44.7%) had R/R AML, and 4 (10.5%) had secondary AML evolving from myelodysplastic syndrome (MDS). The median age of all patients was 67.5 (28–88) years. 60% of the patients were over the age of 65, and 22 (57.9%) were male. According to the ECOG performance score, 10 patients had a performance score of ≤ 1 while 23 (60.5%) had comorbidities. Based on cytogenetic risk classification, 26.3% of the patients were in the favorable-risk group, 15.8% in the intermediate-risk group, and 23.7% in the adverse-risk group; cytogenetic data were unavailable for 34.2%. At the start of treatment, the median blast count for the entire cohort was 37.5% (7–95), and 22 (57.9%) patients had a blast percentage > 30%. Baseline blast percentage refers to the bone marrow blast burden at the time of venetoclax-HMA initiation. In patients with R/R AML, this value may differ from the blast percentage at the time of initial AML diagnosis because of prior AML-directed therapies. Regarding the HMA used with venetoclax, azacitidine was used in 34 (89.5%) patients, decitabine in 1 (2.6%) patient, and both agents sequentially in the remaining 3 (7.9%) patients. Only 11 (28.9%) of all patients received antifungal prophylaxis. No statistically significant differences were found between newly diagnosed and R/R AML patients in terms of age, sex, performance status, comorbidities, baseline blast counts, cytogenetic risk status, the type of HMA agent used, or the use of antifungal prophylaxis (*p* > 0.05). The demographic and clinical characteristics of the patients at the start of treatment, for both the newly diagnosed and R/R AML groups, are shown in Table 1.

**Table 1:** The demographic and clinical characteristics of the patients at the time of treatment

Patients	All cohorts *n* = 38	First line *n* = 12	Second and subsequent line *n* = 26	*p* value
Gender, n (%)				
Male	22 (57.9)	10 (83.3)	12 (46.2)	0.071
Female	16 (42.1)	2 (16.7)	14 (53.8)	
Median age (min-max), months	67.5 (28–88)	68 (53–88)	68 (28–86)	0.299
ECOG performance status, n (%)				
0–1	10 (26.3)	1 (8.3)	9 (34.6)	0.124
2–3	28 (73.7)	11 (91.7)	17 (65.4)	
Comorbidity, n (%)				
Yes	23 (60.5)	9 (75)	14 (53.8)	0.294
No	15 (39.5)	3 (25)	12 (46.2)	
Median baseline blast counts (min-max)	37.5 (7–95)	45 (8–95)	35 (7–95)	0.752
Baseline blast rate, n (%)				
≤ 30%	16 (42.1)	5 (41.7)	11 (42.3)	0.970
> 30%	22 (57.9)	7 (58.3)	15 (57.7)	
Cytogenetic risk, n (%)				
Good	10 (26.3)	2 (16.7)	8 (30.8)	0.529
Intermediate	6 (15.8)	3 (25)	3 (11.5)	
Poor	9 (23.7)	2 (16.7)	7 (26.9)	
Unknown	13 (34.2)	5 (41.6)	8 (30.8)	
HMA, n (%)				
Azacitidine	34 (89.5)	11 (91.7)	23 (88.5)	0.789
Decitabine	1 (2.6)	-	1 (3.8)	
Azacitidine, decitabine	3 (7.9)	1 (8.3)	2 (7.7)	
Fungal prophylaxis, n (%)				
Yes	11 (28.9)	5 (41.7)	6 (23.1)	0.272
No	27 (71.1)	7 (58.3)	20 (76.9)	

Continuous variables are presented as median (min–max), and categorical variables as n (%). Continuous variables were compared using the Mann–Whitney U test. Categorical variables were compared using Fisher’s exact test or chi-square test, as appropriate.

VEN-HMA therapy was used as second-line treatment in 21 (55.3%) patients, while it was used in third or subsequent lines in 5 (13.2%) patients. Among the 26 patients with R/R AML, one patient had relapsed after a previous allogeneic hematopoietic stem cell transplantation (allo-HSCT). The median number of VEN-HMA cycles was 3 (1–18). In the entire patient group, CR was observed in 47.3%, CRi in 21.1%, partial response in 5.3%, and refractory disease in 26.3%. The composite complete remission rate was 68.5% in the entire cohort, while the overall response rate was 73.7%. Post-treatment, 11 (28.9%) patients underwent allo-HSCT. In all patients, the RMST-based PFS was 18.35 months (95% CI 15.16–21.22), and the RMST-based OS was 21.12 months (95% CI 19.03–22.86). Early mortality was evaluated at 30 and 60 days after initiation of venetoclax-HMA therapy. Three patients died within the first 30 days, corresponding to a 30-day mortality rate of 7.9%. By day 60, a total of seven patients had died, resulting in a 60-day mortality rate of 18.4%. There were no statistically significant differences between newly diagnosed AML and R/R AML patients in terms of response rates, overall response rate, proceeding to allo-HSCT after treatment, and OS (*p* > 0.05). However, the median number of cycles administered in newly diagnosed AML patients was statistically significantly higher compared to the R/R AML group [newly diagnosed AML: 5.5 (2–18) vs. R/R AML: 2 (1–18), *p* = 0.002]. A statistically significant difference in OS was observed, with longer survival in the R/R group compared to the newly diagnosed group (23.00 vs. 17.97 months, *p* = 0.024). These comparisons were performed for descriptive purposes only, reflecting the heterogeneous composition of the study cohort, and were not intended to establish comparative treatment efficacy between newly diagnosed and R/R AML. This unexpected finding likely reflects confounding factors including the higher rate of subsequent allo-HSCT in the R/R group (34.6% vs. 16.7%), selection bias inherent to the small sample size, and the retrospective nature of the analysis. This result should be interpreted with considerable caution and does not imply a genuine survival advantage for R/R patients. At the time of the last follow-up, 15 (39.5%) of all patients were still alive and in CR. Post-treatment response evaluation and survival statuses are shown in Table 2.

**Table 2:** Outcomes of venetoclax treatment

	All cohorts *n* = 38	First line *n* = 12	Second and subsequent line *n* = 26	*p* value
Number of treatment line, *n* (%)				
1	12 (31.5)	12 (100)	-	
2	21 (55.3)	-	21 (80.8)	NA
≥ 3	5 (13.2)	-	5 (19.2)	
Median course of treatment (min-max)	3 (1–18)	5.5 (2–18)	2 (1–18)	**0.002**
Treatment response				
CR	18 (47.3)	6 (50)	12 (46.2)	
CRi	8 (21.1)	3 (25)	5 (19.2)	0.779
PR	2 (5.3)	1 (8.3)	1 (3.8)	
No response	10 (26.3)	2 (16.7)	8 (30.8)	
ORR (CR plus CRi plus PR)	28 (73.7)	10 (83.3)	18 (69.2)	0.453
Allo-HSCT after therapy				
Yes	11 (28.9)	2 (16.7)	9 (34.6)	0.444
No	27 (71.1)	10 (83.3)	17 (65.4)	
RMST*-PFS (95% CI), months	18.35 (15.16–21.22)	20.60 (17.05–24.14)	15.33 (10.18–20.49)	0.099
RMST*-OS (95% CI), months	21.12 (19.03–22.86)	17.97 (13.79–22.15)	23.00 (21.69–24.31)	**0.024**

Bold indicates statistically significant results (*p* < 0.05)

*NA* not applicable

* Restricted mean survival time

Response and categorical outcomes were compared using Fisher’s exact test or chi-square test, as appropriate. RMST comparisons were performed using the survRM2 package. RMST was calculated up to 24 months.

**Fig. 1:**
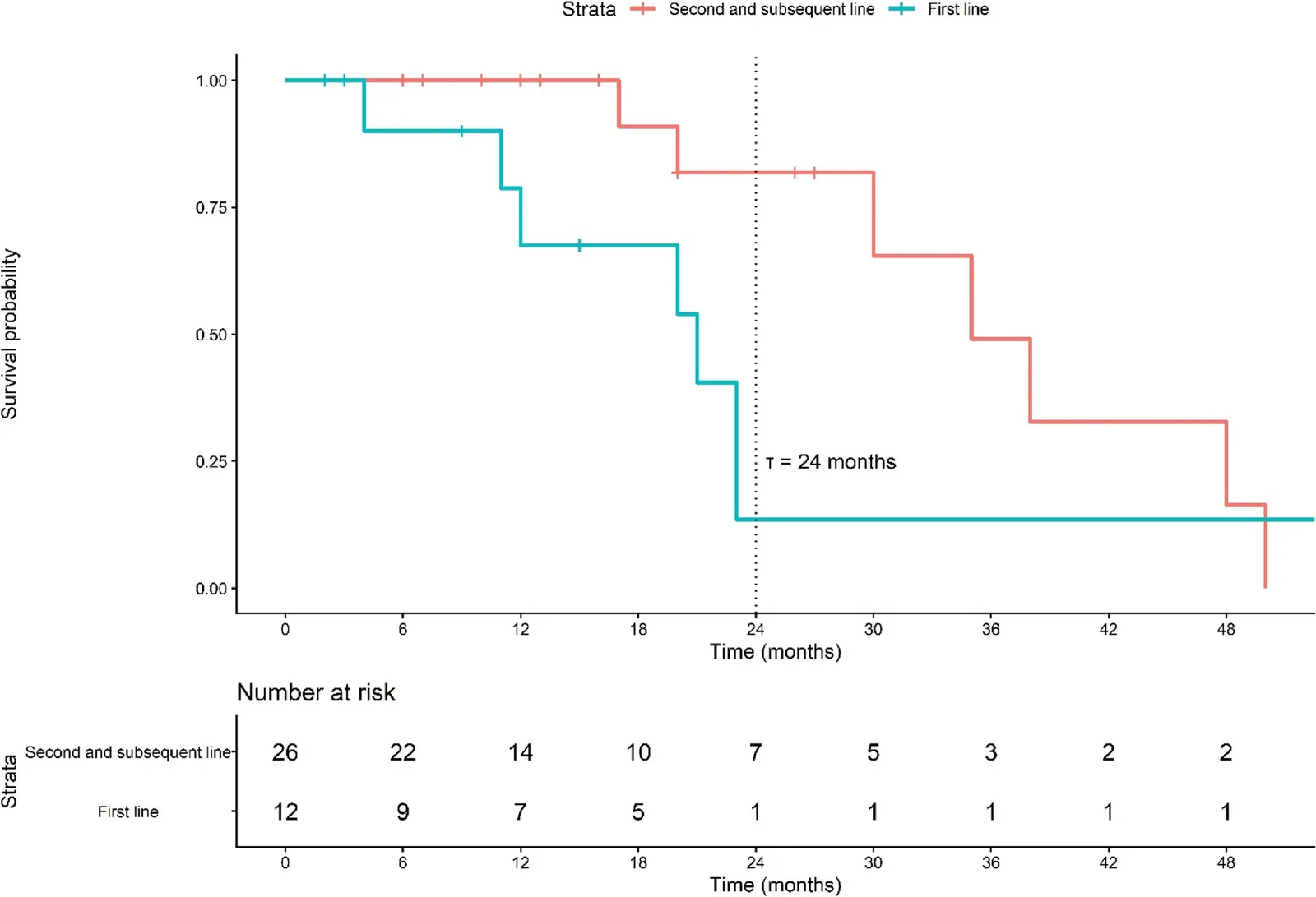
Overall Survival

In the entire cohort, the RMST-based OS was 21.12 months (95% CI: 19.03–22.86). The OS was 17.97 months (95% CI: 13.79–22.15) in patients receiving first-line treatment, whereas it was 23.00 months (95% CI: 21.69–24.31) in those receiving treatment in the second and subsequent lines. A statistically significant difference in OS was observed between those receiving first-line treatment and those receiving treatment in the second and subsequent lines (*p* = 0.024) (Fig. 1).

Although the restricted mean overall survival time was longer in patients treated in the second or subsequent lines, this finding should be interpreted with caution. The survival estimates may have been influenced by the limited sample size, differences in follow-up duration, and potential selection bias inherent to this retrospective cohort. Therefore, these results do not necessarily indicate a survival advantage of later-line treatment over first-line therapy.

**Fig. 2:**
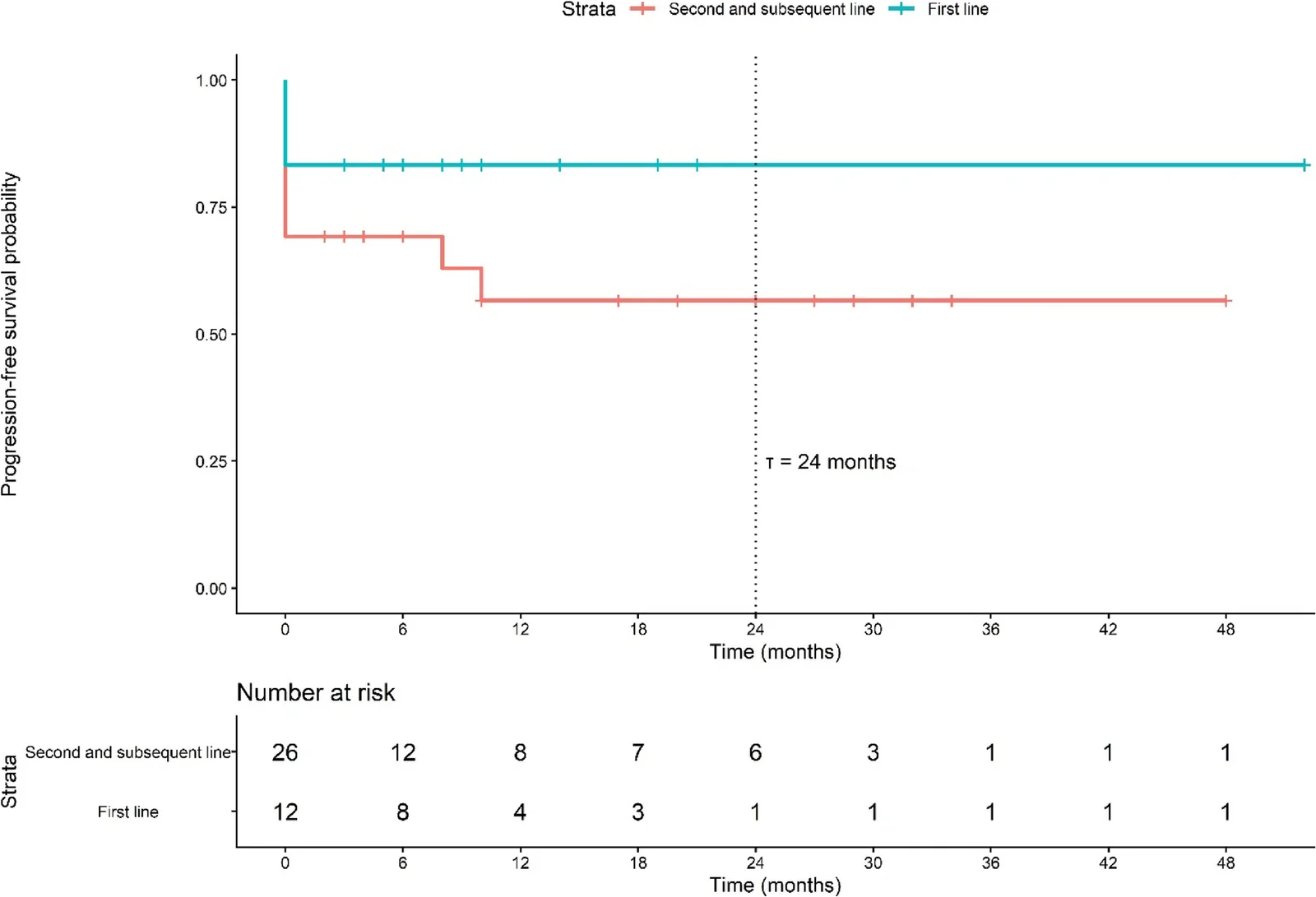
Progression-Free Survival

In the entire cohort, the RMST-based PFS was 18.35 months (95% CI: 15.16–21.22). While the PFS was 20.60 months (95% CI: 17.05–24.14) in patients receiving first-line treatment, it was calculated as 15.33 months (95% CI: 10.18–20.49) in patients receiving treatment in the second and subsequent lines. The difference in PFS between treatment groups did not reach statistical significance (*p* = 0.099) (Fig. 2).

The rate of hematological adverse events was 63.2%, with neutropenia being the most frequent (%52.6). Neutropenia developed as grade 1–2 in 13.1% and grade 3–4 in 39.5% of cases. Thrombocytopenia was observed in 44.8%, with 13.2% at grade 1–2 and 31.6% at grade 3–4. Additionally, neutropenic fever developed in 57.9% of the patients. The adverse event profile of the patients is shown in Table 3.

**Table 3:** Adverse event profile in all patients

Hematological adverse event	*N*:	%
Present	24	63.2
Absent	14	36.8
Neutropenia	20	52.6
Grade 1–2	5	13.1
Grade 3–4	15	39.5
Thrombocytopenia	17	44.8
Grade 1–2	5	13.2
Grade 3–4	12	31.6
Neutropenic fever		
Present	22	57.9
Absent	16	42.1

## Discussion

The present study presents real-life data on HMA-VEN treatment in AML patients. In terms of response rates, the cCR rate was 68%, and the ORR was 73%. As of the last follow-up date, 15 (39.5%) of all patients are still alive in CR. In all patients, the RMST-based PFS was 18.35 months (95% CI 15.16–21.22), and the RMST-based OS was 21.12 months (95% CI 19.03–22.86). When evaluated in terms of adverse events, grade 3–4 neutropenia was found to be 39%, and grade 3–4 thrombocytopenia was found to be 31%.

Venetoclax received approval from the Food and Drug Administration (FDA) with the VIALE-A study. In their study conducted with 431 newly diagnosed patients, there were 286 patients in the aza-ven arm and 145 patients in the aza-placebo (control group) arm. In the response evaluation, a composite complete remission of 66.4% was achieved in the aza-ven arm, while 28.3% was achieved in the control group. At the end of a median follow-up period of 20.5 months, the median OS was 14.7 months in the aza-ven group and 9.6 months in the control group. Safety analyses included grade 3 or higher thrombocytopenia (in 45% and 38%), neutropenia (in 42% and 28%), and neutropenic fever (in 42% and 19%). The aza-ven combination has started to be used as an effective treatment method because it provided significant improvements in the composite complete remission rate and overall survival in elderly AML patients ineligible for intensive chemotherapy. Close monitoring was recommended in the use of this combination due to its myelosuppressive properties [10]. Following the VIALE-A study, real-life data have also begun to appear in the literature [13]. The incidence of neutropenic fever in our cohort (57.9%) was higher than that reported in the VIALE-A trial (42%). This difference may reflect the real-world nature of our cohort, which included a high proportion of patients with ECOG 2–3 performance status (73.7%), a substantial comorbidity burden (60.5%), and non-standardized antifungal prophylaxis practices, all of which may have increased susceptibility to infectious complications. Our finding that only 28.9% of patients received antifungal prophylaxis also reflects the variability of supportive care practices observed in routine clinical practice. A recent nationwide survey from Türkiye reported considerable heterogeneity in antifungal prophylaxis strategies during venetoclax-based therapy, particularly regarding the selection of prophylactic antifungal agents and venetoclax dose modifications required because of CYP3A4-mediated drug interactions [14]. Our findings are consistent with these observations, as posaconazole was the only antifungal agent used for prophylaxis in our cohort, requiring venetoclax dose reduction to 100 mg/day, while most patients did not receive antifungal prophylaxis. These findings highlight the ongoing variability in supportive care practices and underscore the need for greater standardization of antifungal management in patients receiving venetoclax-based treatment.

Following the AVALON study, accumulating real-world evidence has consistently demonstrated that venetoclax-based low-intensity regimens provide clinically meaningful responses in AML patients who are ineligible for intensive chemotherapy [15]. Across published cohorts, composite complete remission rates generally range between 60% and 70% in newly diagnosed patients, while lower response rates are typically observed in the R/R setting. Survival outcomes have also been favorable, with median overall survival commonly reported between 9 and 13 months in newly diagnosed cohorts, although substantial variability exists depending on patient characteristics, disease biology, and access to subsequent allogeneic stem cell transplantation [16–18].

An important observation emerging from real-world studies is the potential role of venetoclax-HMA therapy as a bridge to allo-HSCT. Several cohorts have reported superior outcomes among patients who were able to proceed to transplantation, suggesting that the achievement of remission with venetoclax-based therapy may facilitate access to potentially curative treatment strategies [16]. In addition, comparative studies have shown that venetoclax-based regimens can achieve response rates comparable to, and in some settings higher than, those observed with intensive chemotherapy, while maintaining a more favorable toxicity profile, particularly with respect to severe infectious complications and treatment-related morbidity [19, 20].

The available evidence also indicates that outcomes are generally less favorable in R/R AML than in newly diagnosed disease, reflecting the adverse biological characteristics and treatment resistance commonly encountered in this population [18, 21]. Nevertheless, clinically meaningful responses and prolonged survival remain achievable in a subset of patients, supporting the continued role of venetoclax-based approaches in later treatment lines.

Taken together, the growing body of real-world evidence suggests that the efficacy and safety of venetoclax-HMA therapy are broadly reproducible outside clinical trials despite considerable heterogeneity among patient populations and treatment practices. Differences in response rates, survival outcomes, and toxicity profiles across studies are likely attributable to variations in disease status, cytogenetic and molecular risk features, transplantation rates, supportive care strategies, and follow-up duration. Within this context, further support the feasibility of venetoclax-HMA therapy in routine clinical practice. Nevertheless, it is an undeniable fact that HMA + Ven combination therapy yields survival improvements in this prognostically disadvantaged disease group.

Our results are numerically consistent with — though not directly comparable to — those reported in the VIALE-A trial and larger real-world cohorts, given the substantial differences in sample size, study design, patient selection, and follow-up duration. The observed ORR of 73.7% and OS of 21.12 months should be interpreted as descriptive observations within our specific patient population rather than as confirmatory evidence of efficacy.

Despite the limited sample size, our findings were broadly consistent with those reported in larger real-world cohorts and clinical trials. Therefore, this study provides additional external validation of venetoclax-HMA treatment outcomes in routine clinical practice and supports the generalizability of previously published evidence.

Because our cohort included both newly diagnosed and R/R AML patients receiving venetoclax-HMA therapy, response and survival outcomes were reported separately for descriptive purposes. These analyses were exploratory and were not intended to provide a direct efficacy comparison between these biologically distinct disease settings. The apparent discrepancy between RMST-based PFS and OS estimates in the first-line cohort may be explained by differences in endpoint definitions, as PFS was calculated only among patients who achieved remission after venetoclax-HMA therapy, whereas OS was assessed in the entire treated population from treatment initiation.

Interestingly, patients treated with venetoclax in the second or subsequent lines demonstrated a longer restricted mean overall survival than those treated in the first-line setting. This finding should be interpreted with caution, as it may reflect selection bias rather than a true treatment effect. Patients who survive long enough to receive later-line therapy may represent a subgroup with more favorable disease biology, better performance status, or greater treatment responsiveness. Furthermore, the higher proportion of patients proceeding to allo-HSCT in the R/R group (34.6% vs. 16.7%), together with potential differences in follow-up duration and censoring patterns, may have contributed to the observed survival estimates. In addition, the limited sample size and retrospective nature of the study may have contributed to this unexpected observation. Therefore, these results should not be interpreted as indicating a survival advantage of later-line venetoclax treatment over first-line use and should be considered hypothesis-generating only.

In addition, subsequent allo-HSCT may have contributed to the observed survival outcomes. Future studies with larger cohorts incorporating transplant-censored sensitivity analyses are warranted to better distinguish the effects of venetoclax–HMA therapy from those of subsequent transplantation.

Interpretation of our risk-stratified analyses is further limited by incomplete cytogenetic and molecular characterization of the cohort. In addition to missing cytogenetic data in a proportion of patients, comprehensive molecular information required for contemporary AML risk stratification was not consistently available. Consequently, cytogenetic risk classification was based on ELN 2017 recommendations rather than the updated ELN 2022 criteria. Because several revisions introduced in ELN 2022 depend on molecular abnormalities, our risk categorization may not fully reflect current AML classification standards.

### Study limitation

An important limitation of our study is the relatively small sample size. However, this reflects the real-world nature of the cohort and the highly selected population eligible for venetoclax-HMA therapy at our institution. AML is a relatively uncommon hematologic malignancy, and venetoclax-based treatment became available only during the study period. Furthermore, only patients who were considered unsuitable for intensive chemotherapy and who received at least one cycle of venetoclax-HMA therapy were included. Therefore, the sample size represents the entirety of eligible patients treated at our center during the study period rather than a selectively reduced cohort. Nevertheless, the limited number of patients restricts the statistical power of subgroup analyses and warrants cautious interpretation of the findings. Another limitation of the study is the lack of a contemporaneous comparator cohort treated with alternative non-intensive treatment approaches.

Another important limitation is the incomplete availability of cytogenetic and molecular risk data. Cytogenetic information was unavailable for 34.2% of patients, and comprehensive molecular profiling data, including FLT3, NPM1, IDH1/IDH2, and TP53 mutation status, were not consistently available. Given the well-established prognostic and predictive importance of cytogenetic and molecular abnormalities in AML, these missing data may have introduced residual confounding, limited our ability to fully assess the impact of established risk categories on treatment response and survival outcomes, and precluded detailed molecular risk subgroup analyses. Furthermore, as these abnormalities may influence responses to venetoclax-based therapies, their absence limits the biological interpretation of our findings. Therefore, risk-based analyses should be interpreted with caution. Future prospective studies incorporating comprehensive cytogenetic and molecular profiling with standardized molecular testing are warranted.

Finally, non-standardized antifungal prophylaxis may have resulted in heterogeneous venetoclax exposure due to posaconazole-related dose reductions, potentially affecting efficacy and toxicity outcomes.

## Conclusion

Given the single-center design and small sample size, the findings of this study are hypothesis-generating and should be interpreted accordingly. Within these constraints, our experience suggests that HMA-VEN combination therapy is feasible in routine clinical practice and yields outcomes numerically consistent with larger published cohorts. Although HMA-VEN therapy is now an established treatment approach, real-world data from different patient populations and healthcare settings remain important for confirming the generalizability of outcomes observed in clinical trials.Prospective multicenter studies with molecular profiling are needed to validate these findings and identify predictors of response. The hematologic toxicity profile, including grade 3–4 neutropenia in 39.5% and neutropenic fever in 57.9% of patients, was clinically significant and consistent with the known myelosuppressive effects of this regimen. While these toxicities were manageable in our cohort, close monitoring and individualized supportive care remain essential, particularly in patients with high ECOG performance scores and significant comorbidities.

## Data Availability

The datasets used and/or analyzed during the current study are available from the corresponding author on reasonable request.
